# Contact System Activation in Plasma from Dengue Patients Might Harness Endothelial Virus Replication through the Signaling of Bradykinin Receptors

**DOI:** 10.3390/ph14010056

**Published:** 2021-01-12

**Authors:** Sharton V. A. Coelho, Naiara M. Rust, Lucas Vellasco, Michelle P. Papa, Aline S. G. Pereira, Matheus Ferreira da Silva Palazzo, Maria Aparecida Juliano, Simone M. Costa, Ada M. B. Alves, Marli T. Cordeiro, Ernesto T. A. Marques, Júlio Scharfstein, Luciana B. de Arruda

**Affiliations:** 1 Departamento de Virologia, Instituto de Microbiologia Paulo de Góes, Universidade Federal do Rio de Janeiro, Rio de Janeiro 21941-902, Brazil; shartonvinicius@micro.ufrj.br (S.V.A.C.); naiaramrust@yahoo.com.br (N.M.R.); michelle_premazzi@hotmail.com (M.P.P.); aliine.gomes1@gmail.com (A.S.G.P.); 2 Instituto de Biofísica Carlos Chagas Filho, Universidade Federal do Rio de Janeiro, Rio de Janeiro 21941-902, Brazil; lucasvellasco@hotmail.com (L.V.); matheuspalazzo1@gmail.com (M.F.d.S.P.); 3 Departamento de Biofísica, Escola Paulista de Medicina, Universidade Federal de São Paulo, São Paulo 04023-062, Brazil; ma.juliano@unifesp.br; 4 Laboratório de Biotecnologia e Fisiologia de Infecções Virais, Instituto Oswaldo Cruz, Fundação Oswaldo Cruz, Rio de Janeiro 21040-360, Brazil; simonemc@ioc.fiocruz.br (S.M.C.); ada@ioc.fiocruz.br (A.M.B.A.); 5 Fundação Oswaldo Cruz, Instituto Aggeu Magalhães, Recife 50740-465, Brazil; marli.tenorio@gmail.com (M.T.C.); marques@pitt.edu (E.T.A.M.); 6 Department of Infectious Diseases, University of Pittsburgh, Pittsburgh, PA 15261, USA

**Keywords:** dengue, bradykinin, endothelial cells, kallikrein-kinin system, contact pathway, bradykinin receptor B2

## Abstract

Since exacerbated inflammation and microvascular leakage are hallmarks of dengue virus (DENV) infection, here we interrogated whether systemic activation of the contact/kallikrein-kinin system (KKS) might hamper endothelial function. In vitro assays showed that dextran sulfate, a potent contact activator, failed to generate appreciable levels of activated plasma kallikrein (PKa) in the large majority of samples from a dengue cohort (*n* = 70), irrespective of severity of clinical symptoms. Impaired formation of PKa in dengue-plasmas correlated with the presence of cleaved Factor XII and high molecular weight kininogen (HK), suggesting that the prothrombogenic contact system is frequently triggered during the course of infection. Using two pathogenic arboviruses, DENV or Zika virus (ZIKV), we then asked whether exogenous BK could influence the outcome of infection of human brain microvascular endothelial cells (HBMECs). Unlike the unresponsive phenotype of Zika-infected HBMECs, we found that BK, acting via B2R, vigorously stimulated DENV-2 replication by reverting nitric oxide-driven apoptosis of endothelial cells. Using the mouse model of cerebral dengue infection, we next demonstrated that B2R targeting by icatibant decreased viral load in brain tissues. In summary, our study suggests that contact/KKS activation followed by BK-induced enhancement of DENV replication in the endothelium may underlie microvascular pathology in dengue.

## 1. Introduction

Deregulation of coagulation and fibrinolysis along with an exacerbated inflammatory response are pathological manifestations that are commonly associated to severe viral infections, such as caused by respiratory viruses (Parainfluenza, SARS-CoV, SARS-CoV-2), hemorrhagic-fever viruses (Hantavirus) and Arboviruses [1,2,3,4,5,6]. A major burden to public health in tropical countries, the infection caused by the arbovirus Dengue (DENV) may be relatively mild or severe with increased risk of disease fatality due to hemorrhage, hypotension and hypovolemic shock [5,7,8,9]. Infection by any of the four DENV serotypes (DENV 1–4) can cause these variable clinical signs [10,11,12]. It is estimated that around 50–100 million cases and more than 10,000 deaths occur annually due to dengue infection in approximately 100 countries, with potential for further spread [13]. In Brazil, about 2.3 million dengue cases were registered in 2019 and the number of probable dengue cases increased by almost 19% in the beginning of 2020 summer season [14], coinciding with the period in which the country started to face the consequences of the COVID-19 pandemic. Predictably, symptomatic individuals may be misdiagnosed as dengue, since SARS-CoV-2 infection may also induce thrombocytopenia, in some cases even showing petechiae signals [15,16]. In addition, a case of DENV and SARS-CoV-2 co-infection was reported with severe and lethal neurovascular outcome [17].

It is well established that thrombocytopenia and vascular dysfunction are common manifestations of severe DENV infection. Analysis of human fatal cases and studies in animal models provided evidence that DENV infects endothelial cells lining different tissues [18,19,20,21,22], raising the possibility that microvascular leakage is underpinned by dysregulated endothelial function and/or viral-induced death [23,24,25]. A major limitation in understanding the mechanisms underlining those issues is the lack of an immunocompetent mouse experimental model that sustains virus replication and present dengue clinical signs after systemic inoculation. Since DENV nonstructural proteins 5 (NS5) and 2B/3 (NS2B/3) degrade human, but not mouse STAT2 and STING, respectively, adult mouse animal models poorly reproduce the dengue infection, unless type I and II interferon receptors are knocked out (IFNAR/IFNGR-/-Ag129) [26,27,28]. Therefore, it is still unclear whether systemic thrombo-inflammatory responses might converge with endothelial injury caused by DENV. Nevertheless, intracerebral inoculation of mice is a plausible in vivo model that allows virus replication and measurable clinical parameters [29,30], and have been standardly used in antiviral and vaccine preclinical trials [31,32].

Composed of two serine protease zymogens (FXII and prekallikrein) and the non-enzymatic co-factor high molecular weight kininogen (HK) [33], the contact system promotes thrombo-inflammatory responses [34,35,36,37] by triggering two proteolytic networks: (i) the intrinsic pathway of coagulation and (ii) the kallikrein-kinin system (KKS), a proinflammatory and fibrinolytic cascade [38]. Briefly, activation of the contact system starts when the FXII zymogen interacts with negatively charged surfaces, such as polyphosphates clustered at the surfaces of activated platelets [39]. Once cleaved autocatalytically, FXIIa generates PKa, which in turn reciprocally cleaves FXII. Surpassing the regulatory role of the serpin C1 inhibitor (C1INH), this positive feedback loop rapidly increases the plasma levels of FXIIa and PKa [33]. Acting further downstream, PKa releases the vasoactive bradykinin (BK) nonapeptide from an interior portion of HK. Once liberated, BK and their bioactive metabolites (collectively referred as “kinins”) exert their biological functions by signaling two subtypes of heterotrimeric G-protein coupled receptors (GPCRs): B1R and B2R [40,41]. Unlike B2R, a GPCR that is constitutively expressed by several cell types, B1R is hardly detectable in most healthy tissues. However, B1R transcription in injured tissues is vigorously induced by TNF-α, IL-1 and other proinflammatory cytokines [42,43,44]. Respectively activated by primary kinins (BK/lysil-BK) and kinin metabolites (Des-Arg^9^-BK/Des-Arg^10^-LBK), endothelial B2R and/or B1R share the ability to induce NO-dependent vasodilation and increase microvascular permeability [45]. Once liberated from the kininogens, primary kinins (B2R ligands) may be degraded by angiotensin converting enzyme (ACE) [46] or converted into B1R agonists by carboxypeptidase M [47]. Extent of B1R-driven inflammation is tightly controlled by ACE-2 [48].

Beyond altering microvascular homeostasis via activation of B2R/B1R, kinins stimulate immune resistance against infection by *Trypanosoma cruzi* by upregulating B2R/IL12-dependent Th1 polarization via activation of CD11c dendritic cells [49,50,51]. Typically acting as an opportunistic pathogen, *T. cruzi* takes advantage of B2R-dependent formation of inflammatory edema to fuel intracardiac parasitism [52,53].

Concerning the role of KKS in viral infections, it has been reported that severity of clinical symptoms in patients and experimental models of Rhinovirus, Respiratory syncytial virus (RSV), Influenza virus, and other respiratory viruses correlate with increased kinin levels and kinin receptor expression in nasal secretion and airway epithelial cells [54,55,56]. In vitro studies with Hantavirus provided the first precedent that KKS activation and BK signaling increased endothelial permeability in a viral infection [4]. In a study involving Sindbis virus (SINV), a prototype member of the Alphavirus genus, our group showed that activation of the BK/B2R pathway rendered human microvascular endothelial cells hypersensitive to viral replication [57]. Here we studied the activation profile of contact/KKS in the plasma of a cohort of dengue patients originating from northeast of Brazil. These studies revealed that, in the majority of dengue patients, contact factors are partially activated in the plasma, irrespective of the time-window or clinical severity of the disease. Assuming that the short-lived BK is liberated at the downstream end of the KKS cascade, we then asked whether exogenous kinins could influence the in vitro outcome of endothelial infection. Complementing this work, we resorted to the mouse model of intracerebral model of dengue infection to evaluate whether B2R antagonist (icatibant) has therapeutic potential.

## 2. Results

### 2.1. Bradykinin Increases DENV-2 Replication in Human Microvascular Endothelial Cells

**Figure 1:**
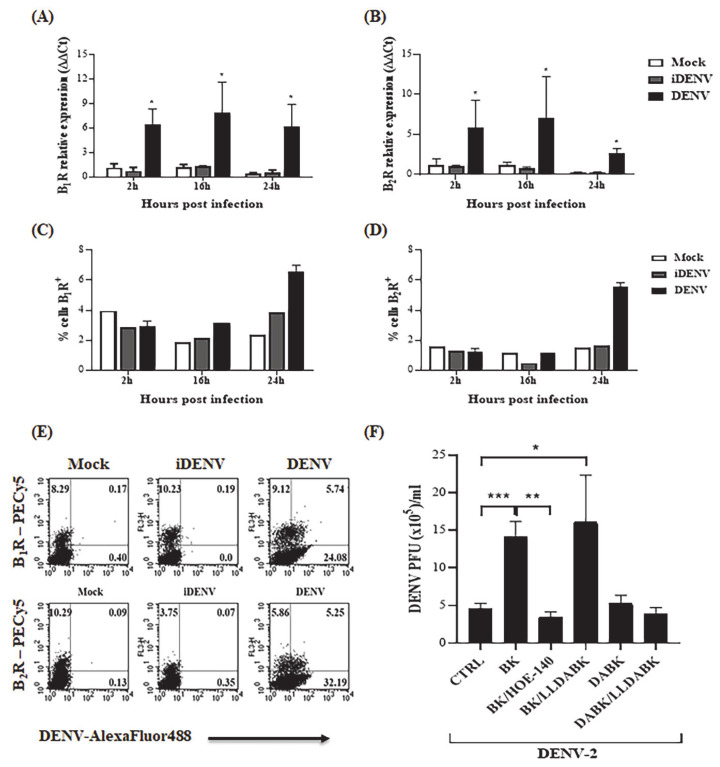
Bradykinin enhances DENV infection in HBMECs in a B2R-dependent way. (**A**–**D**) HBMECs were mock-treated or cultured with DENV-2 in its native or UV-inactivated forms (iDENV) for the indicated time points. (**A**,**B**) The expression of B1R and B2R mRNA were evaluated by qRT-PCR. (**C**,**D**) HBMECs were either incubated with goat anti-B1R antibodies or rabbit anti-B2R (Invitrogen), fixed and stained with secondary anti-goat or anti-rabbit antibodies, respectively. Surface expression of B2R and B1R in HBMECs was measured by flow cytometry; the results are exhibited as the percentage of cells expressing the receptors (% cells), considering staining values obtained in absence of primary antibodies as controls. (**E**) HBMECs cultivated for 24 h in the presence or absence of infectious or inactivated virus as described above were harvested and incubated with rabbit anti-B2R or anti-B1R (Santa Cruz). The cells were then permeabilized and incubated with anti-DENV antibody, followed by incubation with anti-mouse IgG antibody conjugated to AlexaFluor 488. The cells were analyzed by flow cytometry and the figure is a representative quadrant plot of DENV vs. B2R or B1R staining. Insert numbers express the percentage of cells in each quadrant. (**F**) HBMECs were infected with DENV-2 and 24h later, the cells were treated with B2R or B1R agonists-BK or DABK, in the presence or absence of the respective antagonists (HOE-140 and LLDABK). Viral replication was evaluated at 48 h.p.i. by plaque assay. The data are representative of four independent experiments and error bars indicates SD values between replicates in all experiments. * indicates *p* ≤ 0.05; ** *p* ≤ 0.01; *** *p* ≤ 0.001.

Hemostasis dysregulation and exacerbated inflammatory response are thought to contribute to endothelial barrier impairment during severe dengue infection. Using a well-established in vitro model of blood brain barrier [57,58], which was previously demonstrated to be productively infected by DENV serotype 2 (16,681 strain) [59], we first asked whether exogenous kinins added to cultures of human microvascular endothelial cells (HBMECs) could influence the infection outcome. To begin with, we asked whether mRNA levels of B2R and B1R were modulated in HBMECs exposed to infective DENV or UV-inactivated virus. Our results (Figure 1A,B) showed that DENV-2-infected HBMECs upregulated mRNA expression of both GPCRs from 2 h post infection (h.p.i.). Next, we used flow cytometry to analyze the kinetics of the surface expression of BKRs using two different sources of commercial antibodies. In the first series of experiments (Figure 1C,D), we found that surface expression of B1R and B2R was increased at 24 h in DENV-infected HBMECs. Congruent with selective induction observed at transcriptional level, UV-inactivated DENV (iDENV) did not upregulate the expression of these GPCRs above baseline levels, hence linking the functional changes to cumulative virus replication**.** In a second series of flow cytometry experiments, antibody-treated HBMEC (using a second source of anti-BKR antibodies) were permeabilized and treated with mouse anti-DENV IgG. The results (Figure 1E) revealed that a fraction of the virus-infected HBMEC showed upregulated levels of B2R and B1R.

To verify whether signaling of BKRs could modulate DENV-2 replication in HBMECs, we supplemented the culture medium with BK or DABK. Given that the surface expression of BKRs was upregulated at 24 h.p.i., we added the agonists at this time point and evaluated virus replication at 48 h.p.i. Using conventional plaque assay, our results (Figure 1F) showed that addition of BK, but not DABK, significantly increased virus replication in HBMECs. The stimulatory effect of BK was inhibited by the B2R antagonist (HOE-140), whereas the B1R antagonist Lys-[Leu^8^]-Des-Arg^9^-BK (LLDABK) did not interfere with the outcome of infection (Figure 1F). Dose response analysis indicated an inverted U-shape relationship between BK concentration and virus replication (range of 10–200 nM) (Figure S1). Combined, these results revealed that BK, acting via B2R, fuels DENV-2 replication in HBMECs.

**Figure 2:**
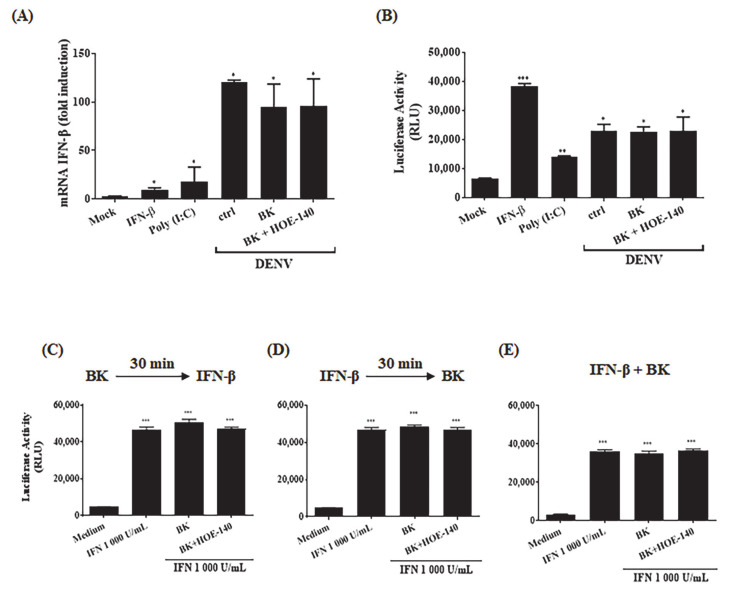
Addition of BK to HBMECs does not interfere with type I IFN response. (**A**,**B**) HBMEC_pISRE luc_ were mock-treated or infected with DENV2. After 24h, the cells were treated with BK, in the presence or absence of HOE-140. At 48 h.p.i., the cells were harvested. As controls, the cells were treated with IFN-β or poly (I:C) for 24h. (**A**) Cell RNA was extracted and analysis of IFN-β mRNA was performed by qRT-PCR. (**B**) The cells were lysed, vortexed, centrifuged and the supernatant were mixed with substrate of luciferase enzyme to analyze the luminescence emission. The data are representative of three independent experiments and error bars indicates SD values between replicates in all experiments. (**C**–**E**) HBMECs were stimulated with IFN-β for 24h. BK was added, in the presence or absence of HOE-140, at different time points, as indicated: 30 min before the addition of IFN-β (**C**), or 30 min after the addition of IFN-β (**D**), or simultaneously to the addition of IFN-β (**E**). The luciferase activity was measured as in (**B**). The data are representative of three independent experiments and error bars indicates SD values between replicates in all experiments. * indicates *p* ≤ 0.05; ** *p* ≤ 0.01; *** *p* ≤ 0.001.

Given that BK was recently reported to inhibit type I IFN responses induced by TLR7 and TLR9 ligands in vivo (normal and lupus-prone mice) and in vitro (human PBMCs) via reduced expression of ISGs and STAT2 phosphorylation [60], we next asked whether BK increased DENV replication in HBMECs by modulating the antiviral type-1 IFN pathway. To this end, we used a reporter HBMEC in which the luciferase gene was regulated by the IFN-stimulated response element (ISRE) promoter (HBMEC_pISREluc_). These transfected cells were infected with DENV-2 and treated with BK (24 h.p.i.) (Figure 2A,B). The expression of IFN-β mRNA and luciferase activity were measured to access type I IFN production and IFN-induced response induced by BK. IFN-β or poly (I: C) were used as positive controls. As predicted [59], the infection of HBMEC with DENV-2 induced the expression of IFN-β (Figure 2A) and activation of ISRE (Figure 2B). However, neither IFN-β mRNA levels nor luciferase activity were modulated by BK, implying that the increase in viral replication did not occur at expense of B2R-dependent down-regulation of IFN-signaling pathways. Consistent with this, addition of BK to uninfected HBMEC cultures (controls) did not affect ISRE response (Figure 2C–E), whether added before, after or simultaneously to IFN-β treatment. Thus, at least for HBMEC in vitro model, there is no evidence that IFN-β responses is suppressed or down-modulated by B2R signaling.

### 2.2. BK Decreases Nitric Oxide Production and Delays Cell Death Induced by DENV-2 in Endothelial Cells

**Figure 3:**
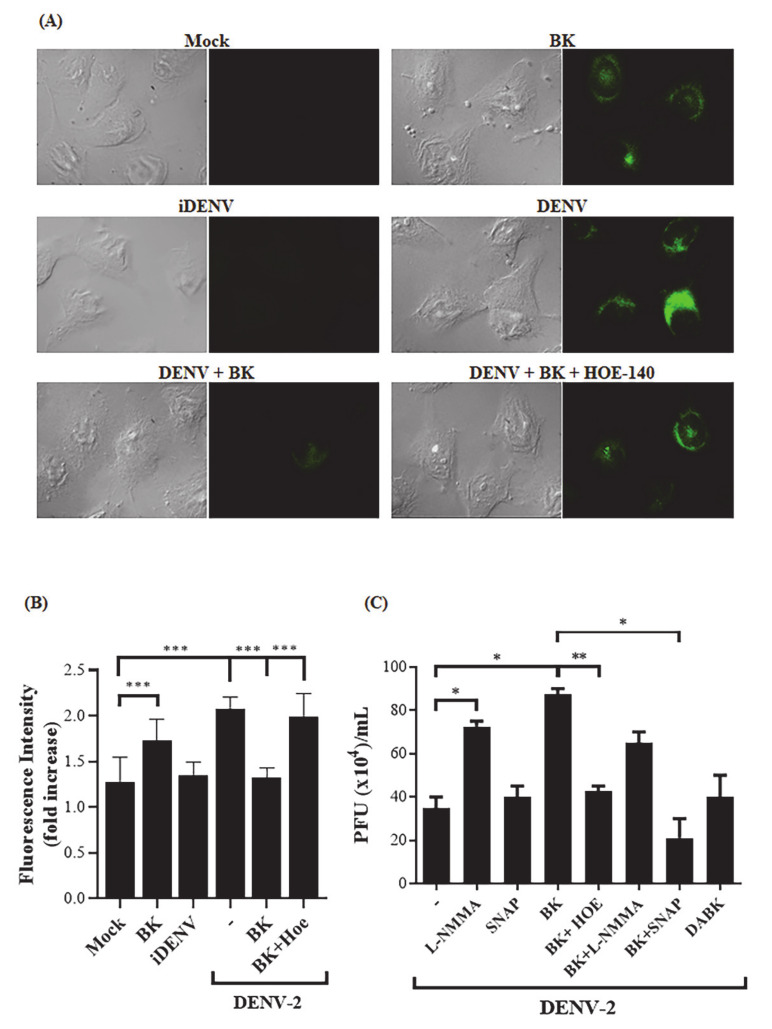
Addition of BK to DENV-infected HBMECs reduced nitric oxide production in a B2R-dependent way. (**A**,**B**) HBMECs were mock-treated or cultured with infectious DENV-2 or UV-inactivated DENV (iDENV). In some cultures, the cells were further incubated with BK for 30 min, in the presence or absence of HOE-140. After 24 h, the cells were incubated with DAF probe. NO production was analyzed by fluorescence microscopy (**A**) and the average of fluorescence intensity was measured by ImageJ software (v. 2.1.0/1.53c) (**B**). (**C**) HBMECs were infected with DENV and 24 h later the cells were treated with BK or L-NMMA, in the presence or absence of HOE-140 or SNAP. At 72 h.p.i., the supernatants were harvested, and viral replication was analyzed by plaque assay. The data are representative of four independent experiments and error bars indicates SD values between replicates in all experiments. * represents *p* ≤ 0.05; ** represents *p* ≤ 0.01; *** represents *p* ≤ 0.001.

One of the main events triggered by B2R signaling is the regulation of nitric oxide (NO) production [61,62], which is also induced by DENV replication in different cell types [63,64,65]. Awareness that NO exerts antiviral effects in vitro and in vivo [12,66,67] led us to consider the possibility that BK could interfere in NO production induced by DENV infection. To address this question, DENV-2-infected HBMECs were treated with BK (24 h.p.i.), in the presence or absence of HOE-140, and NO levels were measured by immunofluorescence. As predicted, control experiments showed that DENV-2 and BK individually stimulated NO production in HBMECs (Figure 3). Interestingly, however, the addition of BK to DENV-2-infected HBMECs (24 h.p.i.) significantly reduced the intracellular levels of NO. Notably, NO inhibition by BK was counteracted by the addition of HOE-140 (Figure 3A,B). These data suggest that activation of the B2R signaling pathway in DENV-2-infected HBMECs downregulated NO production and raised the possibility that impaired NO production renders HBMECs increasingly susceptible to viral replication. To test the hypothesis that BK inhibited the anti-viral response of NO, DENV-2-infected HBMECs were treated with BK in the presence or absence of SNAP NO donor and virus replication was accessed by plaque assay (Figure 3C). As a control, infected HBMECs were cultured with the nitric oxidase inhibitor L-NMMA. Controls showed that addition of L-NMMA to DENV-2-infected HBMECs increased virus production. Notably, the addition of SNAP to BK-treated cultures hampered BK-mediated increase of virus replication and the virus titers were similar to those detected in cultures devoid of exogenous BK (Figure 3C). These results suggest that BK, otherwise a classical inducer of NO production by healthy endothelial cells, suppresses NO production in DENV-2-infected HBMECs, ultimately rendering the stressed endothelial cells increasingly susceptible to viral replication.

**Figure 4:**
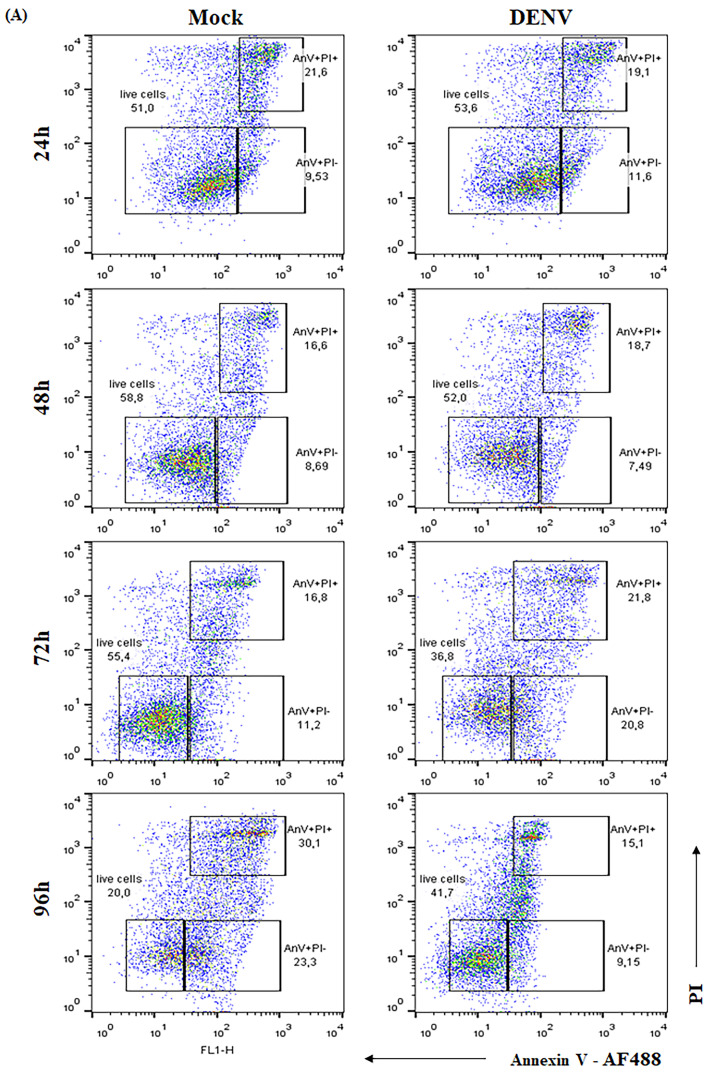

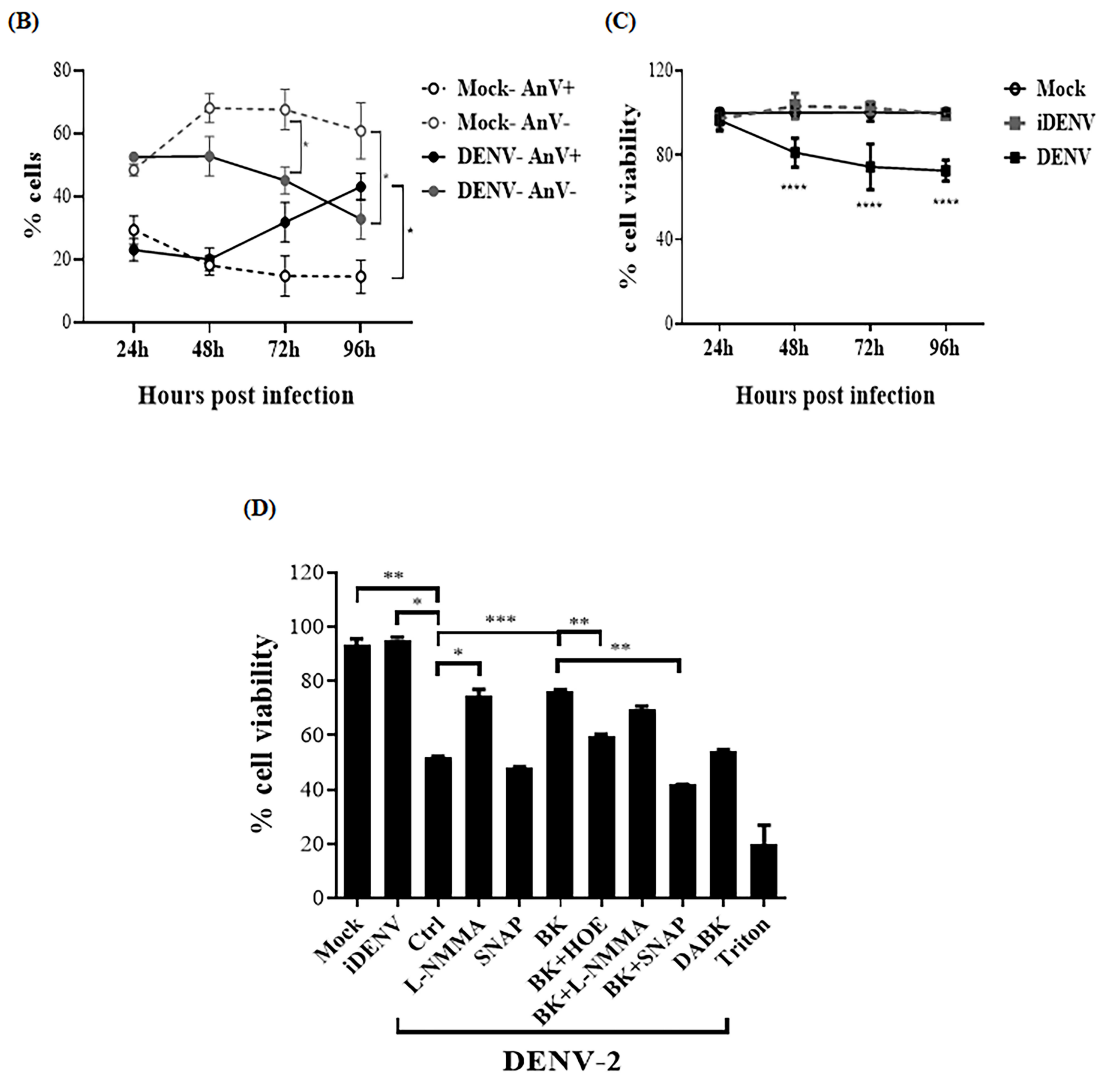
B2R activation and NO inhibition promotes a delay in DENV-induced cell death. (**A**,**B**) HBMECs were mock-treated or infected with DENV and, after different time points, the cells were stained with Alexafluor488-conjugated annexin V and propidium iodide (PI) and the frequency of apoptotic cells (% AnV+) was analyzed by flow cytometry. A representative dot blot is indicated in (**A**) and the average frequency of AnV+ and AnV- cells obtained in two independent experiments in indicated in (**B**). (**C**) HBMECs were mock-treated or cultured with DENV-2 in its native or inactivated forms (iDENV) and cell viability was evaluated by XTT metabolization assay at the indicated time points. (**D**) HBMECs were mock-treated or cultured with DENV-2 or iDENV for 24h and then, the cells were treated with BK or L-NMMA, in the presence or absence of HOE-140 or SNAP. At 72 h.p.i., cell viability was accessed by XTT metabolization assay. The data were normalized according to the results obtained with the cells cultured with culture medium only (adjusted to 100%). The data are representative of four independent experiments and error bars indicates SD values between replicates in all experiments. * represents *p* ≤ 0.05; ** *p* ≤ 0.01; *** *p* ≤ 0.001; **** *p* ≤ 0.0001.

Apoptosis is a common cellular strategy to control virus dissemination, and prolonged cell survival may result in higher viral loads [57,68,69]. In vitro studies with endothelial cells have previously linked the B2R activation and NO pathway to modulation of cellular proliferation and apoptosis [57,70,71]. Since DENV-2 infection is known to induce HBMECs cell death [59], we sought to determine whether this anti-viral response was suppressed as due to activation of the B2R/NO pathway. Initially, we evaluated HBMEC viability upon DENV-2 infection by AnnexinV/PI double staining and flow cytometry (Figure 4A,B) and by XTT metabolization assay (Figure 4C). Decreased cell survival started to be detected at 48 h.p.i. in both assays, and increased apoptosis phenotype was significantly detected at 72 h.p.i. (Figure 4A,B). UV-inactivated DENV-2 did not affect cell viability. We then stimulated DENV-2-infected HBMEC with BK or L-NMMA and found that both treatments rescued the cells from death (*p* < 0.001) (Figure 4D). Importantly, the antiapoptotic effect of BK in DENV-infected HBMEC was reversed by the addition of HOE-140 or SNAP (Figure 4D).

**Figure 5:**
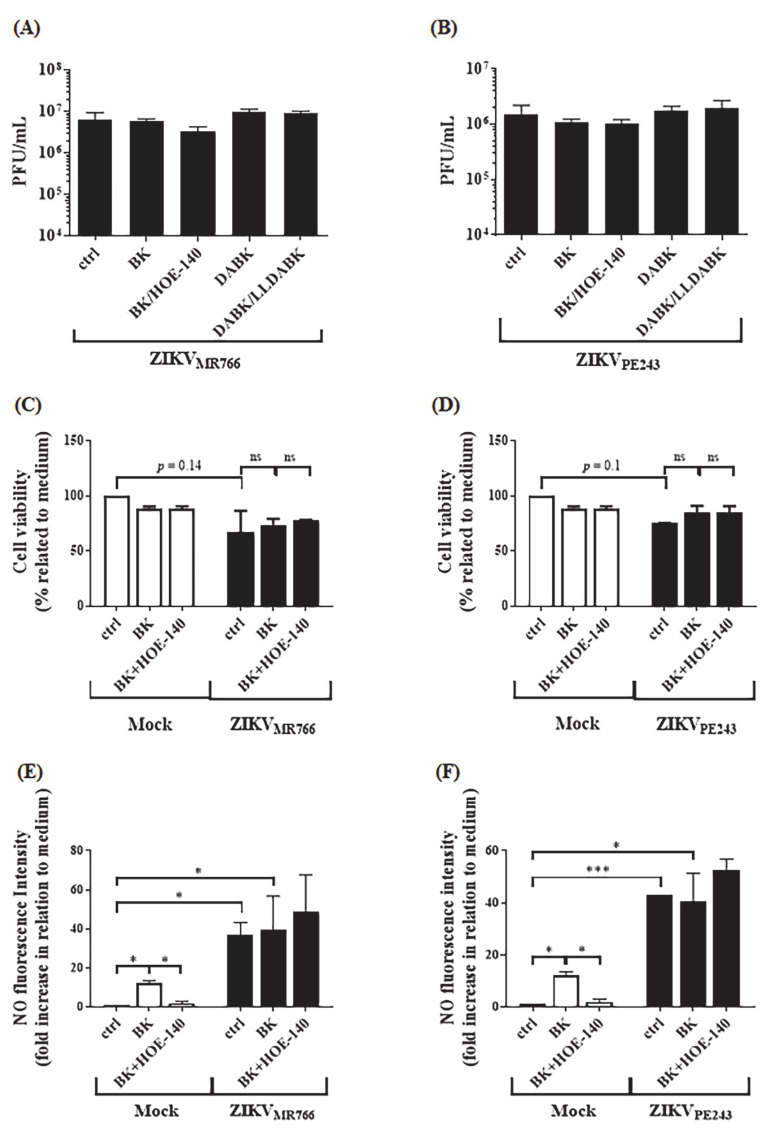
Bradykinin does not affect ZIKV replication. HBMECs were mock-treated or infected with ZIKV_MR766_ (**A**,**C**,**E**) or ZIKV_PE243_ (**B**,**D**,**F**). (**A**,**B**) At 24 h.p.i., the cells were treated with BK or DABK, in the presence or absence of the B2R or B1R antagonists-HOE-140 or LLDABK, respectively. Viral replication was evaluated at 48 h.p.i. by plaque assay. (**C**–**F**) At 24 h.p.i., ZIKV-infected HBMECs were treated with BK in the presence or absence of HOE-140. At 48 h.p.i., cell viability was assessed by XTT metabolization assay (**C**,**D**) and NO production was measured by immunofluorescence after DAF probe staining (**E**,**F**). The data are representative of three independent experiments. * represents *p* ≤ 0.05; *** *p* ≤ 0.001.

To verify whether BK also favored endothelial infection by other flaviviruses, we repeated the same experimental designs with HBMECs pre-infected with two different strains of Zika virus (ZIKV), representatives of African and Asian lineages (ZIKV_MR766_ and ZIKV_PE243_). Intriguingly, addition of BK or DABK to HBMEC-infected cultures did not modulate ZIKV replication (Figure 5A,B), nor did BK reduced cell viability or NO production (Figure 5C–F).

### 2.3. Ex Vivo Evaluation of the Contact Pathway Activation in Plasmas Obtained from Dengue Patients

**Figure 6:**
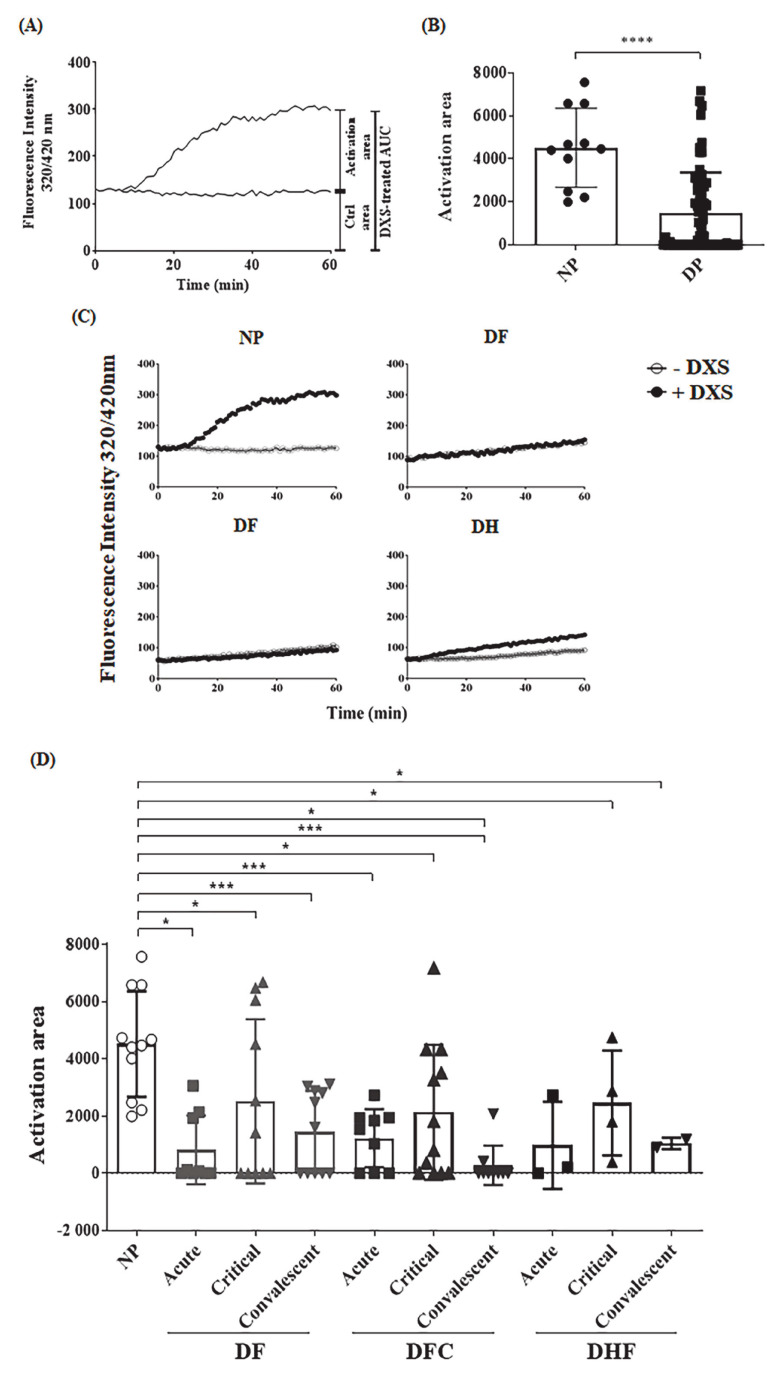
Plasmas from DENV-positive patients present lower PKa activation when challenged with DXS**.** Plasmas obtained from dengue patients (DP) or control donors (NP) were incubated with intramolecularly quenched peptide substrate (PKaS), in the presence or not of dextran sulfate (DXS). Activation of KKS was evaluated by measuring fluorescence emission due to substrate hydrolysis for 1 h. (**A**) Representative curve obtained after spectrofluorimetric analysis (O.D.) with normal human plasmas (NP) incubated or not with DXS. The subtraction of the control area (ctrl) from the total area represent the activation area, which will be used in the bar graphs. (**B**) Activation area graphs obtained after analysis of the hydrolysis assays. Bars represent mean ± SD, and the dots indicate the individual data obtained from all NP in comparison to all plasmas from dengue patients (DP). (**C**) Representative hydrolysis curves obtained with one NP and DP from patients clinically classified as dengue fever (DF), complicated dengue fever (DFC) or dengue hemorrhagic fever (DHF) treated or not with DXS. (**D**) Plasmas samples were collected at acute (2–3 days), critical (5–7 days) and convalescent phases (<30 days) of infection from the same patients. The plasmas were incubated with PKaS, in the presence or absence of DXS. PKa activity was evaluated in each sample by measuring the substrate hydrolysis and the activation area was measured based on the hydrolysis plot. * represents *p* ≤ 0.05; *** *p* ≤ 0.001; **** *p* ≤ 0.0001.

Although kinins can be generated via multiple proteolytic pathways, there is a consensus that the main bradykinin-forming enzyme in the bloodstream is plasma kallikrein (PKa), a serine protease generated by FXIIa, the initiator of contact system activation. In previous studies, we measured PKa activity in human or animal plasma following incubation of contact activators using intramolecularly quenched fluorogenic substrates that span the flanking sides of bradykinin in human HK [52,72]. Using this enzymatic assay, we checked whether dextran sulfate (DXS) was able to generate PKa in plasma samples from a cohort from Pernambuco, Brazil, collected from 11 healthy donors (normal human plasmas–NP) and from 27 dengue patients (dengue human plasmas–DP). The patients were clinically classified as dengue fever (DF), complicated dengue fever (DFC), or dengue hemorrhagic fever (DHF). Some of the plasmas analyzed here were collected at different time points after diagnosis. Based on the time point after onset of dengue symptoms, the plasma samples were subclassified as acute (2–3 days, *n* = 21), critical (5–7 days, *n* = 27) or convalescent (>20 days, *n* = 22) phase of infection, totaling 70 plasma samples. The hematological parameters, time of sample collection and clinical classification of each patient are described in Table 1. As explained in methods, the temporal progression of PKa activity in DXS-treated and untreated plasmas varied from one patient to another. To overcome this problem, we measured the area under the curve for DXS-treated plasma and subtracted from the area obtained with the same plasma with no DXS stimulation, establishing individual activation areas (Figure 6A). Internal controls included assays with heat-inactivated plasma (iPlasma) or fresh plasma samples supplemented with the synthetic PKa inhibitor PKSI-527 (Figure S2A). Also, since the plasmas were collected from patients infected in geographically distant endemic areas and were stocked in ultrafreezers, we conducted a separate control study to evaluate whether freezing of the plasmas would affect PKa generation. For this purpose, we collected normal healthy plasmas, froze them for at least a week, and thawed at the moment of the analysis, which was run in parallel with freshly collected plasmas (Figure S2B,C). To further validate contact phase screening with DXS, we compared PKa activity in plasmas collected with low molecular weight heparin or with citrate and found that the extent of hydrolysis (area under the curve) was not significantly altered (Figure S2D,E).

Having established the area under the curves as a criteria for measuring PKa activity in plasma activated or not by DXS, we then screened samples from different clinical forms of dengue as well as plasma from dengue-negative donors from the same cohort. Inspection of the hydrolysis curves (Figure 6B–D and Figure S3) revealed that DXS-treated plasma from dengue patients display lower levels of PKa as compared to normal plasma (NP), irrespective of severity of clinical disease. We then performed a longitudinal evaluation by assaying PKa activity in plasma samples from the same donors, yet collected at the acute, critical and convalescent phases. Our results (Figure 6D and Figure S3) showed decreased PKa activity in DXS-treated plasma irrespective of the clinical stage of dengue.

### 2.4. DENV Modulates the Kallikrein-Kinin Pathway by Consuming Plasma Elements Early after Infection

**Figure 7:**
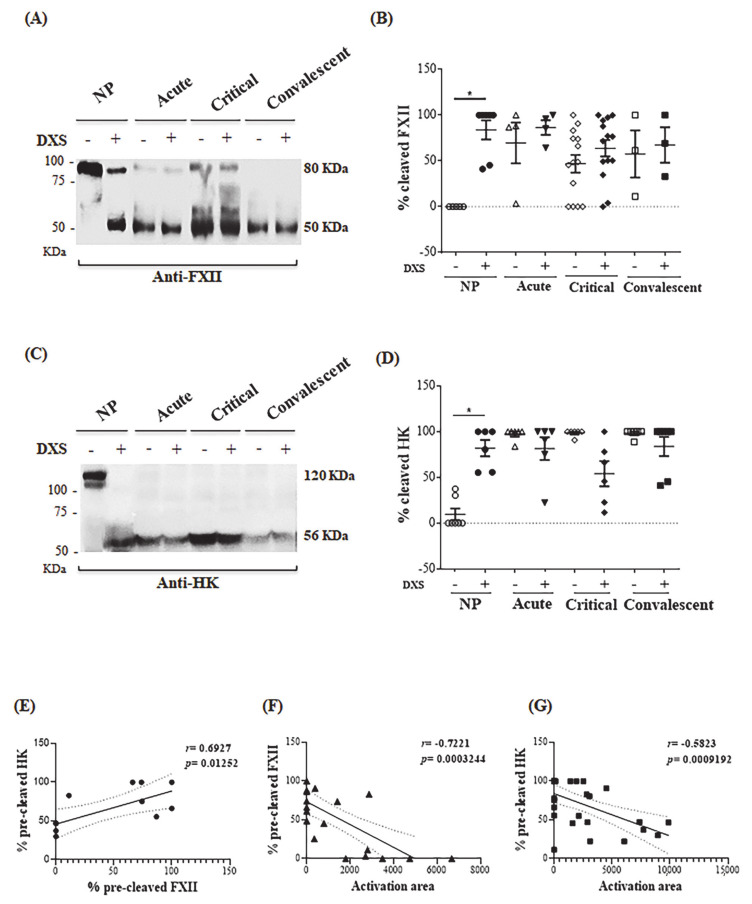
Evidence of early consumption of FXII and HK in the plasmas obtained from DENV-positive patients. NP or DP, at different stages of infection, were incubated or not with DXS for 1 h, at 37 °C. The presence of FXII and HK were evaluated by western blotting. The bands corresponding to intact and cleaved proteins were measured using ImageJ software and the percentage of cleaved proteins was calculated. (**A**) Representative western blotting of FXII levels in plasmas collected from a single patient, at different time points of infection; (**B**) Individual plots of the percentage of cleaved FXII from plasmas incubated or not with DXS; (**C**) Representative western blotting of HK levels in plasmas collected from a single patient, at different time points of infection. (**D**) Individual plots of the percentage of cleaved HK from plasmas. * indicates *p* ≤ 0.05. (**E**) Correlation graph and calculation between the frequency of cleaved FXII and cleaved HK, in the absence of DXS (pre-cleaved), as measured by western blotting. (**F**) Correlation graph and calculation between PKa activation, as measured by the hydrolysis assay (activation area) with the frequency of cleaved FXII, in the absence of DXS (%pre-cleaved FXII), measured by western blotting; (**G**) Correlation graph and calculation between PKa activation, as measured by the hydrolysis assay (activation area) with the frequency of cleaved HK, in the absence of DXS (%pre-cleaved HK), measured by western blotting.

Given the evidence that DXS-treatment of dengue plasma failed to induce full-blown activation of PKa, we then carried western blotting to evaluate whether contact factors (FXIIa, HK) were activated. Western blotting revealed high levels of cleaved FXII and HK in the majority of dengue plasmas patients. Noteworthy, dengue plasma displayed FXIIa and HKa irrespective of addition of DXS, and the profile did not change as the infection progressed (Figure 7A–D). In contrast, plasma from normal donors (NP) from the same cohort showed intact forms of FXII and HK. As expected, addition of DXS to NP converted the zymogens into FXIIa and HKa. As demonstrated in the hydrolysis assays, plasma freezing for a few days before the assay did not affect the integrity of FXII and HK nor the generation of FXIIa and HKa after DXS stimulation (Figure S2F–I). We then measured the proportion of pre-cleaved FXII and HK levels (without DXS stimulation) in individual plasma samples from our cohort and compared with the PKa activity generated upon incubation with DXS (activation area). Using this parameter, DXS-inducible PKa activity was negatively correlated with cleaved FXII and HK chains (*p* < 0.001). As predicted, we found that the extent of cleavage of FXII and HK chains were positively correlated (*p* = 0.01) (Figure 7E–G). These results suggest that impaired generation of PKa in DXS-treated plasma is a consequence of in vivo activation of plasma contact factors during the course of dengue infection. We next checked whether plasma levels of C1INH (glycosylated or non-glycosylated forms) differed between DPs and NPs. Although increased levels of C1INH in the plasmas of dengue patients had been previously reported [73,74], we did not detect appreciable variability in the levels of C1INH in our cohort (Figure S4A–C)

In another report, Puerta-Guardo and colleagues [75] showed evidences that DENV induces endothelial dysfunction via activation pathways mediated by non-structural protein 1 (NS1). Using the PKa assay, purified NS1 did not induce contact activation even when the concentration was raised to 12 μM, which is approximately 1000-fold higher than NS1 concentration found in the plasma of severe dengue patients [76,77] (Figure S4D). Additionally, since the plasma levels of ferritin and LDL are strongly increased during dengue infection [78,79], we added these purified factors to normal plasma and verified that they did not inhibit contact system activation by DXS (Figure S4E,F).

### 2.5. B2R Targeting in the Intracerebral Model of Dengue Reduced Viral Load in Brain Tissues

**Figure 8:**
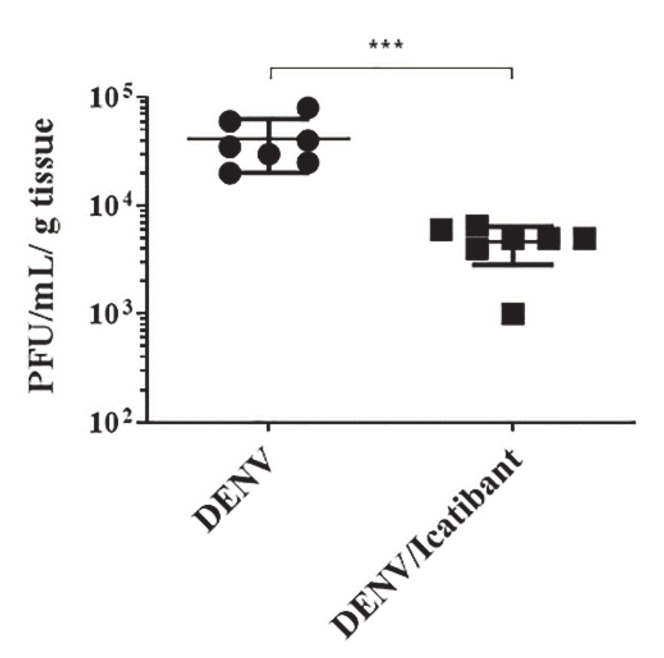
In vivo treatment with the B2R antagonist icatibant reduced DENV titer in the brain of wild type mice. 8-week-old Balb/c mice were infected with DENV NGC, i.c., in the presence or absence of icatibant. At 3dpi, the animals were euthanized, and the brains were harvested for viral load evaluation by conventional plaque assay. Bars represent mean ± SD and dots indicate individual mice. *** *p* ≤ 0.001.

In this section, we resorted to an intracerebral model of dengue infection in mice to evaluate whether targeting of endothelial B2R may hamper DENV replication in brain tissues. This question was addressed using Balb/c mice (8 weeks old) infected with a neuroadapted DENV-2 strain (NGC) [29,30], inoculated with or without the B2R antagonist icatibant. At 72 h post inoculation, the brains were collected, and viral replication was evaluated by plaque assay. Our results showed that blockade of B2R signaling with icatibant significantly decreased the viral load in the brain tissues (Figure 8). Although the cellular targets of DENV in brain tissues are unknown, these results indicate that this arbovirus might coopt the KKS to increment its infectivity via BK-induced activation of endothelial B2R.

## 3. Discussion

KKS activation in the settings of viral infections may have broader implications to health, from hemorrhagic to respiratory diseases. Increased bradykinin levels were observed in nasal secretion of rhinovirus and influenza A challenged individuals and positively correlated with the clinical outcome [56,80]. A direct role of virus replication in sensitivity to BK stimuli was evidenced by upregulated expression of BKR in infected epithelial cells, an effect ascribed to innate sensing of dsRNA [55]. Edelman and collaborators [81] were the first to report that Thai children with DHF presented significantly decreased plasma levels of FXII and PK when compared to donors with unrelated fever. Focusing on the regulation of serine protease cascades, others have found increased plasma levels of C1INH in dengue patients as compared to healthy controls [73].

In a survey of plasmas from 27 dengue patients, here we report that DXS failed to activate the contact system (PKa activity as read-out) in the majority of dengue plasma, irrespective of the time window and clinical form of the disease. Unlike results reported elsewhere [8], we found that plasma levels of C1INH were not upregulated in the dengue-cohort, perhaps reflecting differences in the clinical end-points of these studies (DF vs. DHF) or comparison between NP vs. DP. Also, other technical strategies should be used to measure C1INH in a future study to completely exclude its role in the effect. We then screened PKa activity in our dengue cohort and found a negative correlation with the extent of FXII and HK cleavage. Noteworthy, in a limited study involving six DHF samples, we did not find any correlation between altered contact activity either with platelet counts or with transaminases levels, but this topic warrants further investigation with a larger cohort.

Since the large majority of dengue patients did not present severe symptomatology, we may infer that the partial activation of the pro-thrombogenic contact system in dengue patients is not per se sufficient to impair endothelial barrier function in vivo. This is in accordance with independent studies showing that inflammatory and coagulation pathways are activated early in dengue infections irrespective of severity of bleeding [7]. The finding that HK is cleaved to a variable degree in the majority of our dengue samples suggests that BK might have been liberated in the systemic circulation of dengue patients. Although BK levels in the plasma were not measured at point of care, it is conceivable that patients with mild symptomatology do not suffer microvascular abnormalities because the liberated BK is swiftly degraded by ACE and/or other metallopeptidases [82,83].

Although the complement system is classically viewed as a serine protease cascade that integrates inflammation to immunity, this concept has been revised to incorporate coagulation as a key component of intravascular immunity [84,85]. Involving reciprocal activation of leukocytes and platelets, this primitive mechanism of host defense is thought to promote microbial retention in fibrin networks via cooperative activation of the extrinsic and intrinsic pathways of coagulation. Whether involving polyphosphates clustered at the surfaces of platelets or NETS liberated by activated neutrophils, our study suggests that the contact system (FXII/PK/HK) is activated during the course of dengue infection. The first precedent of a virus that manipulates the KKS as a strategy to increment its infectivity came from our studies of endothelial infection by the arbovirus Sindbis [57]. Extending the breadth of these early studies, in the present work we demonstrate that BK increases endothelial susceptibility to infection by DENV without interfering with the outcome of infection by ZIKV, another arbovirus that cause severe pathology in humans.

Given the technical obstacles to investigate the impact of KKS activation on endothelial dysfunction in adult animals, we chose to examine the outcome of infection in HBMEC, a line of human microvascular (brain) endothelial cells that was extensively used in studies of parasite trans-migration through the brain-blood barrier [58]. After showing that HBMECs are susceptible to infection by the DENV serotype-2, we verified that B2R and B1R transcription were rapidly upregulated.

Unlike the phenotype of ZIKV, we showed that BK robustly increased (i) the load of DENV via activation of endothelial B2R (ii) host cell survival by diminishing formation of nitric oxide via activation of B2R. As expected, we found that both BK and DENV (added separately) stimulated NO production in non-infected HBMECs. Although the antiviral effect of NO was previously described [66,86,87], we were surprised that BK inhibited NO production in infected HBMECs because B2R signaling classically upregulates NO production by normal endothelial cells via activation of different isoforms of NO synthase (eNOS) [88,89,90,91]. This seemingly contradictory results may be explained by a study showing that BK, acting via B2R expressed by human coronary endothelial cells, increased the production of NO, which in turn down-regulated eNOS [92]. Although not mutually exclusive, it will be interesting to determine whether Sindbis and DENV (unlike ZIKV) subvert the B2R signaling pathway to inhibit NO-dependent apoptosis by targeting eNOS via NOSIP, a regulatory protein that normally protects endothelial cells from NO-induced damage during the G2 phase of the cellular cycle [93].

Intriguingly, we found that addition of lysyl-DABK (B1R agonist) to DENV-2-infected HBMECs did not increase viral replication. Future studies may clarify whether the unresponsive B1R phenotype of DENV-infected HBMECs might reflect differential regulation of eNOS by B2R versus B1R signaling pathway [88]. Alternatively, it will be interesting to determine whether upregulated expression of ACE-2 in DENV-infected HBMECs may account for the unresponsive phenotype of B1R agonist [48]. Although the role of B1R and ACE-2 in dengue infection remains unknown, knowledge stemming from pathogenesis of coronaviruses (SARS-CoV and SARS-CoV-2) comes to mind because these viruses elicit thrombo-inflammatory responses while infecting cell types that overexpress the ACE-2 surface receptor [3,94,95]. Pertinently, a first fatal case of stroke associated to severe neurovascular damage/ischemic lesions was reported in a Brazilian patient co-infected with DENV and SARS-COV-2 [17]. Given that dengue is endemic in most regions of Brazil, it will be worth evaluating whether KKS activation in patients with double infections may further increase risk of COVID-19 fatalities.

Since BK increased the viral load in cultures of HBMECS, a line of microvascular endothelial cells derived from human brain, we sought to evaluate whether B2R targeting was protective in a mouse model of intracerebral infection established with DENV-2 [29,30]. In a previous study, we demonstrated that after 5 to 7 days of intracerebral infection with DENV there is perivascular infiltration of inflammatory lymphocytes in choroid plexus and perivascular areas, associated to hemorrhagic foci [30]. Using icatibant, a B2R blocker that efficiently reduce swelling episodes in patients with type-1 and acquired hereditary angioedema patients [96,97], this pharmacological intervention reduced viral load in brain tissues of mice challenged with an extremely high dose of virus (40 LD_50_). Efforts along these lines have yielded promising results in the first non-randomized trials conducted with icatibant in cohorts of COVID-19 patients [98]. However, as note of caution, one must be born in mind that BKR agonists are known to increase blood brain barrier permeability [99], therefore, it is conceivable that icatibant may have reduced the extent of inflammation inflicted by the surgical procedure. Disruption of the blood brain barrier may also facilitate the spread of DENV viruses in the subendothelial matrix, allowing for viral-induced degranulation of perivascular mast cells [100]. Although conceding that the intracerebral model of dengue infection in mice has limitations (does not present the systemic complications associated to severe dengue infection), and that we have limited the end-point to analyses of viral load in the brain, our findings should encourage efforts to investigate the therapeutic potentialities of B2R/B1R antagonists in arboviruses leading to hypovolemic shock and thrombo-inflammation.

## 4. Material and Methods

### 4.1. Ethical Statements

Plasma samples were obtained from a well-controlled dengue cohort established in Recife (Pernambuco, Brazil) [101]. The dengue clinical cohort was reviewed and approved by the ethics committee of the Brazilian Ministry of Health (CONEP: 4909; Process 25000.119007/2002-03; CEP: 68/02 and CONEP 12138) and by the Institutional Review Board of the University of Pittsburgh (PRO10020398). Each suspected dengue case (or his/her guardian) in the dengue clinical cohort provided written informed consent and all data were anonymized. Additional blood samples from deidentified dengue negative donors were obtained from the Hemotherapy Service at the Hospital Universitário Clementino Fraga Filho (HUCFF) of Universidade Federal do Rio de Janeiro (UFRJ). The study protocol was approved by the Experimental Ethics Committee of UFRJ (Permit Number: 105/07).

Balb/c mice were maintained at the mice facility of the Instituto Oswaldo Cruz (IOC), Fundação Oswaldo Cruz (Fiocruz), Brazil. The animals were housed according to institutional policies for animal care and usage and the protocol was approved by The Ethics Committee of Animal Care and Use from Instituto Oswaldo Cruz (CEUA-IOC) (Permit Number: no L022/2019).

### 4.2. Cells and Virus

Human brain microvascular endothelial cells (HBMEC) [58], kindly given by Dr. Dennis J. Grab, The Johns Hopkins University, Baltimore, MD, USA) were cultured in medium 199 (M199-Invitrogen, Carlsbad, CA, USA) supplemented with 10% of inactivated fetal calf serum (FCS-Invitrogen). Baby hamster kidney cells (BHK-21 [C-13]; ATCC^®^ CCL-10^™^) were cultured in Minimum Essential Medium Eagle-Alpha Modification (α-MEM) (Invitrogen) supplemented with 5% of FCS. African green monkey kidney cells (Vero cells; ATCC^®^ CCL81) were cultured in M199. *Aedes albopictus* clone C6/36 (ATCC^®^ CRL-1660^™^) cells were cultured in Leibovitz (L-15) medium (Invitrogen), supplemented with 10% FCS. Laboratory strains of DENV-2, 16,681 Asiatic strain and mouse adapted New Guinea C (NGC) (GenBank M29095), were propagated in C6/36 and Vero cell lines, respectively. ZIKV strain MR766 (ZIKV_MR766_; ATCC VR1838) was kindly given by Dr. Amilcar Tanuri (Instituto de Biologia, UFRJ, RJ), and ZIKV strain PE243 (ZIKV_PE423_, gene bank ref. number KX197192) was isolated at Centro de Pesquisas Aggeu Magalhães, FIOCRUZ, PE, Brazil). Both ZIKV strains were propagated in C6/36 cells. The supernatants of infected cells were harvested, filtered and stored at −80 °C. DENV-2 virus stocks were titrated by plaque assay in BHK cells, whereas ZIKV stocks were titrated using Vero cells, as previously described [57,102]. The supernatants obtained from noninfected cell lines were used as mock control. Inactivated virus (iDENV) was obtained after U.V. exposition for 2 h; inactivation was confirmed by plaque assay and RT-PCR.

### 4.3. HBMEC Infection and Stimulation In Vitro

HBMECs (10^5^ cells/mL) were incubated overnight for cell adhesion and, when achieved 60–70% confluence, cells were cultured with DENV-2 (16,681 strain), ZIKV_MR766_ or ZIKV_PE243_, at a multiplicity of infection (MOI) of 1. Supernatants of noninfected C6/36 cells (mock) or UV-inactivated DENV (iDENV) were used as controls. After 90 min of incubation at 37 °C, cells were washed with endotoxin-free PBS and cultured with M199 with 10% FCS. At 24 h post infection (h.p.i.), the cells (around 90% confluency) were treated or not with 10nM of bradykinin (BK) (Sigma-Aldrich, St. Louis, MO, USA) or Lys-Des-Arg^9^-bradykinin (DABK) (Sigma-Aldrich) after 30 min of preincubation with lisinopril (25 µM) (Sigma-Aldrich), an inhibitor of the angiotensin converting enzyme (ACE). At some experimental points, the cells were also treated with the antagonist of B2R-HOE-140; or with the antagonist of B1R Lys-[Leu^8^]-Des-Arg^9^-BK (LLDABK; 100 nM; (Sigma-Aldrich)); or with the inhibitor of nitric oxide synthase (NOS) L-NMMA (250 µM; Sigma-Aldrich); or with the nitric oxide donor SNAP (150 µM; Sigma-Aldrich). The antagonists and inhibitors were added 30 min before the addition of the BK or DABK, when indicated. The cells and supernatants were harvested at 48 h.p.i. and virus infection was analyzed by plaque-forming assay, or flow cytometry.

### 4.4. Virus Titration

HBMECs were mock-treated or infected with DENV-2, in the presence or absence of BK or other stimuli, as described. The supernatants obtained from these cultures were harvested at the indicated time points and the concentration of infectious DENV particles was measured by plaque assay, as described [57].

For in vivo experiments, the brains were collected, weighed using a precision scale and macerated in RPMI medium (Invitrogen) in the ratio of 1 μL of medium for each 0.2 mg of tissue. Then the macerate was centrifuged at 400× *g* for 5 min and the supernatant used for titration by conventional plaque assay. The results were normalized to PFU/g of tissue.

### 4.5. Evaluation of BKR Expression by qRT-PCR and Flow Cytometry

HBMECs were mock-treated or cultured with DENV-2 in its native or UV-inactivated form (iDENV) and harvested at 2, 16 and 24 h.p.i. To analyze the expression of B1R, B2R mRNA, total RNA was extracted from the cells using the TRIzol reagent (Thermo Fisher Scientific Inc., Waltham, MA, USA), according to the manufacturer’s instructions. Total RNA (1 μg) was reversely transcribed to cDNA using a High-Capacity cDNA Archive kit (Thermo Fisher Scientific Inc.), following the parameters: 25 °C for 10 min, two steps of 37 °C for 60 min and 85 °C for 5 min and chilled in −20 °C. The cDNA was subjected to real-time PCR using Power SYBR Green PCR master mix reagent (Thermo Fisher Scientific Inc.), with the following primers: B1R sense (5′- GGG CCG CAA GGA TAG CAA GAC-3′), B1R antisense (5′- GCA GGC CCA GGT CAA TGA AGT-3′), B2R sense (5′-ACA CAG GGC AGT CAT TCA GCA C-3′), B2R antisense (5′-CAT GCA TAG GGC CGC TCT TC-3′), GAPDH sense (5′-GTG GAC CTG ACC TGC CGT CT-3′), and GAPDH antisense (5′-GGA GGA GTG GGT GTC GCT GT-3′). The reactions were performed in duplicates and were carried out in a StepOnePlus real-time PCR system (Thermo Fisher Scientific Inc.). The samples were subjected to 50 °C for 2 min, 95 °C for 10 min, and 40 cycles, with 1 cycle consisting of denaturation (95 °C, 15 s), primer annealing (55 °C, 30 s), and primer extension (60 °C, 1 min). Melting curve analysis was used to eliminate primer dimers: 95 °C for 15 s, 60 °C for 1 min, and 95 °C for 15 s. The comparative *C_T_* method (ΔΔCt) [103] was used to quantify gene expression levels with human GAPDH (Applied Biosystems, Waltham, MA, USA) assay for normalization, once all assays met the amplification efficiency criteria of 100% ± 10% (Applied Biosystems - Application Note 127AP05-02).

To analyze the expression of B1R and B2R protein the cells were incubated with PBS with 10% BSA for 30 min for blocking. The cells were then incubated with goat anti-B1R or rabbit anti-B2R antibodies (5 μg/mL; Invitrogen/Thermo Fisher Scientific Inc.) for 30 min, followed by 1h incubation with secondary antibodies conjugated to AlexFluor488 and fixed with 4% formaldehyde for 10 min. In some experiments, the cells were stained with rabbit anti-B1R or anti-B2R (Santa Cruz Biotechnologies, Santa Cruz, CA, USA), followed anti-rabbit IgG conjugated to PECy5. The cells were, then, fixed and permeabilized with 0.1% saponin, and incubated with mouse anti-DENV type II antibody (clone 3H51; Chemicon/Merck Millipore, Darmstadt, Germany), followed by incubation with anti-mouse IgG antibody conjugated Alexa fluor-488 (Life Technologies, Carlsbad, CA, USA). Samples stained in absence of primary antibodies were used as controls. The samples were analyzed in a FACScalibur cytometer, using FlowJo software (v10.7.1; Becton Dickson Immunocytometry System).

### 4.6. Evaluation of IFN-β Production and IFN-Mediated Response

Analysis of IFN-β production and IFN-mediated response were performed using a reporter HBMEC, in which the *interferon stimulated response element* (ISRE) promoter is linked to a luciferase gene (HBMECpISREluc; kindly given by Dr. Laura Gil, CPqAM, FIOCRUZ, Recife, PE, Brasil). The cells were mock-treated or infected with DENV-2 (16,681 strain, at a MOI of 1). After 24h, the cells were cultured with BK (100 nM) and lisonopril (25 μM–30 min), in the presence or absence of HOE-140 (100 nM). As controls, the cells were only stimulated with IFN-β (1000 U/mL) or poly (I: C) (50 μg/μL) for 24h. At 48 h.p.i., the cells were harvested, and the RNA was extracted, using TRIzol reagent, according to manufacturer’s protocol (Thermo Fisher Scientific Inc.), and first strand cDNA was synthesized using High-Capacity cDNA Archive Kit (Applied Biosystems), according to the manufacturer’s instructions. Expression of IFN-β mRNAs was measured with SYBR Green (Applied Biosystems), using the following primers: IFN-β sense: 5′-TAG CAC TGG CTG GAA TGA GA-3′, IFN-β antisense 5′ TCC TTG GCC TTC AGG TAA TG -3′; GAPDH sense 5’-GTG GAC CTG ACC TGC CGT CT-3’ and GAPDH antisense 5’-GGA GGA GTG GGT GTC GCT GT-3’. The samples were subjected to the following reaction: 50 °C for 2 min, 95 °C for 10 min and 40 cycles of denaturation (95 °C, 15 s), primer annealing (55 °C, 30 s), and primer extension (60 °C, 1 min). Next, the samples were subjected to a melt curve to eliminate primer dimers: 95 °C, 15 s; 60 °C, 1 min and 95 °C, 15 s. Comparative CT method (ΔΔCt) was used to quantify gene expression levels with GAPDH used for normalization.

IFN response was evaluated by measuring the luciferase activity in infected and/or treated cells. HBMECs were infected with DENV-2 and treated as described before. In another set of experiments, the cells were only treated with IFN-β in the presence or absence of BK, with or without HOE-140. At 24 h post stimuli, the cells were washed with PBS and lysed using 200 μL/well of Luciferase Cell Culture Lysis 1X Reagent-CCLR reagent (Promega, Madison, WA, USA) following the manufacturer’s recommendations. The lysate was vortexed for 10 s and centrifuged at 6000 rpm for 2 min at 4 °C, and the supernatant collected for luminescence assay preparation. 20 μL of cell lysate supernatant was mixed with 50 μL of Luciferase Assay Reagent (Promega) enzyme substrate in a 96-well plate (GloMax-Promega), and luminescence was measured on a GloMax^®^-Promega luminometer according to the recommendations of the manufacturer. Results were presented in relative luminescence units (RLU).

### 4.7. Nitric Oxide Production

HBMECs (5 × 10^4^ cell/mL) were mock-treated or infected with DENV-2 (16,681 strain, at a MOI of 1), in its native or inactivated (iDENV) forms. After 24 h, the cells were incubated with DAF-FM probe (Invitrogen) for 30 min, followed by another 30 min in serum free culture. In some cultures, the cells were further incubated with lisinopril, followed by 30 min incubation with BK, in the presence or absence of HOE-140 (100 nM). Nitric oxide production was then analyzed by fluorescence microscopy and the average fluorescence intensity was measured by ImageJ software.

### 4.8. Cell Viability

HBMEC (10^5^ cell/mL) were mock-treated or infected with DENV-2 (16,681 strain, MOI 1). At different time points post infection (24–96 h), the cells were incubated with AlexaFluor488-conjugated Annexin V and propidium iodide and the frequency of apoptotic cells was analyzed by flow cytometry. In another set of experiments, cell viability was evaluated at the same time points after infection by XTT metabolization assay, according to manufacturer’s protocol (Abcam, Cambridge, MA, USA), as previously described [104]. To investigate the role of BKR signaling and NO modulation, at 24 h.p.i., the cells were treated with BK, HOE-140, L-NMMA or SNAP, as indicated. HOE-140, SNAP and L-NMMA were added 30 min before the addition of BK, when indicated. At 72 h.p.i., cell viability was evaluated by XTT metabolization assay.

### 4.9. DENV Plasmas Samples Cohort

In this study we used plasmas obtained from well-controlled cohort of dengue patients from Pernambuco, Brazil [101]. Dengue cases were clinically classified according to the World Health Organization (WHO) criteria from 1997 [105] into dengue fever (DF) or severe dengue hemorrhagic fever (DHF). Dengue cases presenting thrombocytopenia but not fulfilling the requirements for DHF were classified as complicated dengue fever (DFC). Blood was collected using low molecular weight heparin. Conventional serological methods to measure IgM and IgG, PCR, and virus isolation were performed in all samples for diagnosis, serotyping and classification of the patients as primary or secondary dengue. Hemogram, leukogram, analysis of plasma proteins and hepatic enzymes were also measured. Table 1 indicate all the virological and hematological parameters and the classification of the samples analyzed in this study. Some patients had blood collected at different periods of time after symptom onset. The samples obtained from the same patient were grouped in the table and time of blood harvesting was indicated. Plasmas from donors diagnosed as dengue negative were collected and maintained in the same conditions and classified here as control normal human plasmas (NP).

### 4.10. Ex Vivo Analysis of Contact System Activation

Plasmas from dengue patients or control donors were incubated with an intramolecularly quenched kallikrein substrate flanking the BK sequence of human kininogen (PKaS; Abz-G-F-S-P-F-R-S-S-R-I-Q-EDDnp; 4 µM). The plasmas were then treated or not with the contact activator DXS (500 kDa; 20 nM). Inactivated plasmas, or addition of the plasma kallikrein inhibitor PKSI-527 (200 μM; kindly given by Dr. Luis Juliano, UNIFESP, SP, Brazil) were used as assay controls. The reaction was carried out in PBS, pH 7.4 and the hydrolysis of PKaS was monitored by spectrophotometry for 1 h, as previously described [72]. The O.D. values obtained were plotted in a graph x/y (time/fluorescence) and the area of the graph was calculated based on the curve inclination obtained. The activation area was then calculated by subtracting the area obtained with the plasmas treated with DXS from the area of hydrolysis yielded by untreated plasma from the same donor (area under the curve) (Figure 6A). Since the plasmas were collected from patients infected in geographically distant endemic areas and were stocked in ultrafreezers, additional controls were separately conducted by comparing previously frozen and freshly collected normal healthy plasmas. To further validate contact phase screening with DXS, we also compared PKa activity in plasmas collected with low molecular weight heparin or with citrate [52].

### 4.11. Evaluation of the Role of NS1, Apoferritin and LDL

Increasing concentrations of NS1 (Abcam; ab64456), apoferritin (Sigma-Aldrich) and LDL (Sigma-Aldrich) were added to healthy plasma samples in the presence or absence of DXS (20 nM). Activation of the kallikrein-kinin pathway was measured by spectrophotometric hydrolysis assay and represented by the activation area as previously described.

### 4.12. Analysis of FXII, HK and C1INH by Western Blotting

Normal human plasmas (NP) or dengue human plasmas (DP), at different stages of infection, were incubated or not with DXS for 1 h, at 37 °C and subsequently the samples were reduced at a final concentration of 710 mM β-mercaptoethanol in Laemmli Sample Buffer (Bio-Rad, Hercules, CA, USA) and denatured for 5 min at 95 °C. Then, 40 µg of the different plasma samples were subjected to 8% polyacrylamide gel electrophoresis (SDS-PAGE) followed by transfer to nitrocellulose membrane. Anti-FXII (Santa Cruz–1:500), anti-HK (Santa Cruz 1:500) and anti-C1INH (Santa Cruz 1:500) were used to detect cleavage and glycosylation profile (inferred by molecular weight). The bands corresponding to intact and cleaved proteins were measured using ImageJ software, and the percentage of cleaved and glycosylation ratio by non-glycosylation proteins was calculated.

### 4.13. Mouse Infection

Male BALB/c mice specific pathogen free (SPF) of 8-week-old, purchased from the Multidisciplinary Center for Biological Research (CEMIB, Campinas/SP) were inoculated by the intracerebral (i.c.) route with 30 μL of a neuroadapted dengue virus serotype 2 (DENV2) NGC, which corresponds to 2.04 log10 PFU/mL and approximately 40 LD_50_. Virus samples were diluted in M199, mixed or not with 100 μg/Kg of icatibant (Fyrazir, Lexington, MA, USA), an antagonist of B2 receptor. Before i.c. inoculation and treatment, animals were anesthetized with a mixture of ketamine-xylazine (100 mg/Kg ketamine; 10 mg/Kg xylazine) and three days p,i. they were overexposed to this mixture for euthanasia. The brains were collected, and viral load were analyzed using plaque assay in BHK cells. The results were represented by PFU/g of tissue.

### 4.14. Statistical Analysis

Data were analyzed using the GraphPad Prism software (GraphPad Software v.8.4.0, San Diego, CA, USA). Comparisons among conditions were performed by *t* test; *p* < 0.05 were considered statistically significant.

## 5. Conclusions

In conclusion, we demonstrated that activation of B2R in endothelial cells potentiated DENV replication by hampering the production of NO, thereby inhibiting NO-dependent host cell death. In addition, ex vivo studies with plasma from a limited cohort revealed that DENV infection leads to activation of the contact/plasma kallikrein-kinin pathway. Future studies may clarify whether BK, acting as a fuel of DENV replication in endothelial cells, may worsen the clinical outcome of Dengue infection.

## Figures and Tables

**Table 1:** Dengue fever, complicated dengue fever, and dengue hemorrhagic fever patients. All samples were positive for DENV3 PCR.

Patient Code	Gender	Age	Phase of Infection	Days of Symptoms	IgM	IgG	Leukocytes	Platelets	Hematocrit	Albumin	Denv Clinical Classification	Infection
Patient 1			Acute	4	+	−	3420	337,000	33.4	4.3		
	F	8	Critical	8	+	+	6190	362,000	31.95	4.3	DF	Primary
			Convalescent	29	+	+	9840	497,000	34.9	4.6		
Patient 2			Acute	4	−	+	nd	nd	nd	nd		
	M	47	Critical	7	−	+	6530	189,000	41.7	nd	DF	Secondary
			Convalescent	36	+	+	nd	nd	nd	nd		
Patient 3			Acute	3	−	−	nd	nd	nd	nd		
	M	38	Critical	5	−	−	nd	nd	nd	nd	DF	Primary
			Convalescent	48	+	+	nd	nd	nd	nd		
Patient 4			Acute	4	+	+	nd	nd	nd	nd		
	F	37	Critical	6	+	+	5100	248,000	38	4.1	DHF	Secondary
			Convalescent	18	+	+	nd	nd	nd	nd		
Patient 5												
	F	12	Critical	6	−	−	nd	nd	nd	nd	DF	Primary
			Convalescent	26	+	+	nd	nd	nd	nd		
Patient 6												
	M	35	Critical	8	+	−	6270	125,000	45.8	3.8	DHF	Primary
												
Patient 7												
	F	33	Critical	5	−	−	1760	110,000	36.4	4.2	DFC	Primary
			Convalescent	37	+	+	nd	nd	nd	nd		
Patient 8			Acute	3	−	+	4900	134,000	39.4	3.3		
	M	41	Critical	9	+	+	4420	131,000	46.8	3.4	DHF	Secondary
												
Patient 9			Acute	4	+	−	2400	52,000	43.5	3.9		
	F	15	Critical	6	+	−	2500	66,000	37.9	nd	DFC	Primary
												
Patient 10			Acute	3	−	+	3080	105,000	38.8	nd		
	M	48	Critical	5	−	+	2580	92,000	42.4	4.2	DFC	Secondary
			Convalescent	38	+	+	4970	152,000	40.9	nd		
Patient 11			Acute	3	−	−	nd	nd	nd	nd		
	M	23	Critical	5	−	−	nd	nd	nd	nd	DF	Primary
			Convalescent	30	+	+	nd	nd	nd	nd		
Patient 12			Acute	3	−	+	nd	nd	nd	nd		
	F	30	Critical	5	−	+	nd	nd	nd	nd	DF	Secondary
			Convalescent	31	−	+	6410	166,000	38.2	4.4		
Patient 13			Acute	3	−	+	nd	nd	nd	nd		
	F	40	Critical	5	−	+	3000	120,000	41.4	4.13	DFC	Secondary
			Convalescent	33	−	+	nd	nd	nd	nd		
Patient 14			Acute	3	−	+	3700	124,000	47.3	nd		
	M	31	Critical	5	−	+	2900	120,000	46	4.48	DFC	Secondary
			Convalescent	18	−	+	9700	283,000	43.4	4.54		
Patient 15			Acute	4	−	+	2360	143,000	35.7	3.8		
	F	40	Critical	7	+	+	3810	143,000	34.3	4.0	DF	Secondary
			Convalescent	30	−	+	5040	327,000	32.6	nd		
Patient 16			Acute	4	+	+	5750	14,000	39.1	nd		
	M	45	Critical	6	+	+	5200	110,000	41.7	4.2	DFC	Secondary
			Convalescent	34	+	+	nd	nd	nd	nd		
Patient 17			Acute	3	−	+	4440	125,000	45.2			
	M	44	Critical	5	+	+	3060	107,000	44.9	4.5	DFC	Primary
			Convalescent	34	+	+	7290	185,000	45.9	4.7		
Patient 18			Acute	4	−	+	2600	116,000	41.2	nd		
	F	47	Critical	6	+	+	3500	92,000	38	nd	DFC	Secondary
			Convalescent	33	+	+	6700	505,000	38.4	nd		
Patient 19			Acute	3	−	−	nd	nd	nd	nd		
	M	42	Critical	5		−	nd	nd	nd	nd	DF	Primary
			Convalescent	31	+	+	nd	nd	nd	nd		
Patient 20			Acute	3	+	+	2770	211,000	41.3	nd		
	F	53	Critical	5	+	+	3260	164,000	39	4.2	DF	Secondary
			Convalescent	41	+	+	nd	nd	nd	nd		
Patient 21												
	F	27	Critical	6	+	−	nd	nd	nd	nd	DF	Primary
			Convalescent	33	+	+	nd	nd	nd	nd		
Patient 22			Acute	4	−	+	2100	90,000	34.6	3.7		
	F	21	Critical	6	+	+	nd	nd	nd	nd	DF	Secondary
			Convalescent	48	+	+	nd	nd	nd	nd		
Patient 23												
	M	18	Critical	6	−	−	nd	nd	nd	nd	DFC	Primary
												
Patient 24												
	F	49	Critical	8	−	+	5910	178,000	33.6	3.9	DFC	Secondary
			Convalescent	30	−	+	nd	nd	nd	nd		
Patient 25			Acute	4	−	+	3560	131,000	40.2	3.9		
	M	48	Critical	7	−	+	8260	114,000	40.3	3.6	DFC	Secondary
												
Patient 26			Acute	4	−	+	1260	133,000	34.7	3.5		
	F	31	Critical	8	+	+	3600	141,000	34.4	nd	DFC	Secondary
			Convalescent	52	+	+	nd	nd	nd	nd		
Patient 27			Acute	3	−	+	4770	122,000	42.8	4.2		
	M	36	Critical	6	+	+	2170	71,000	47.2	3.9	DHF	Secondary
			Convalescent	37	+	+	nd	nd	nd	nd		

nd, not determined; white, light gray and dark gray indicate plasma samples collected at acute, critical of convalescent phases of infection, respectively.

## Data Availability

The data presented in this study are available in the compressed files.

## Supplementary Materials

The following are available online at https://www.mdpi.com/1424-8247/14/1/56/s1, Figure S1: BK dose response of DENV increased replication in HBMEC, Figure S2: Evaluation of the interference of the plasmas stock condition for the hydrolysis assay., Figure S3: Hydrolysis of all DENV samples at different time points upon the symptom’s onset., Figure S4: Lower DXS-driven KKS activation in the plasmas from dengue patients is not due to C1inhibitor, NS1, ferritinemia, or increased LDL.
