# Supplementary material for: Contact System Activation in Plasma from Dengue Patients Might Harness Endothelial Virus Replication through the Signaling of Bradykinin Receptors
Source: Pharmaceuticals (Basel). 2021 Jan 12;14(1):56. doi: 10.3390/ph14010056 (PMC7827195; doi:10.3390/ph14010056)
Supplement: Supplementary file 1 [file pharmaceuticals-14-00056-s001.pdf]

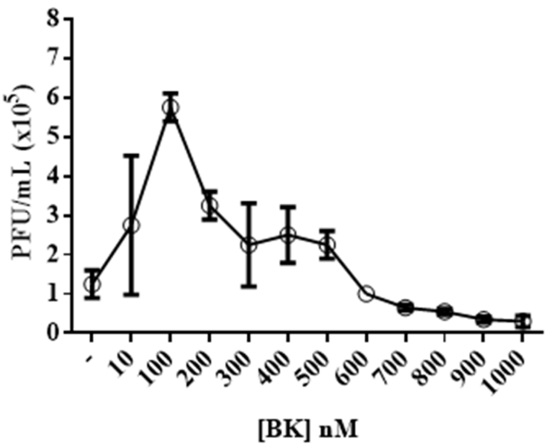

Figure S1. BK dose response of DENV increased replication in HBMEC.

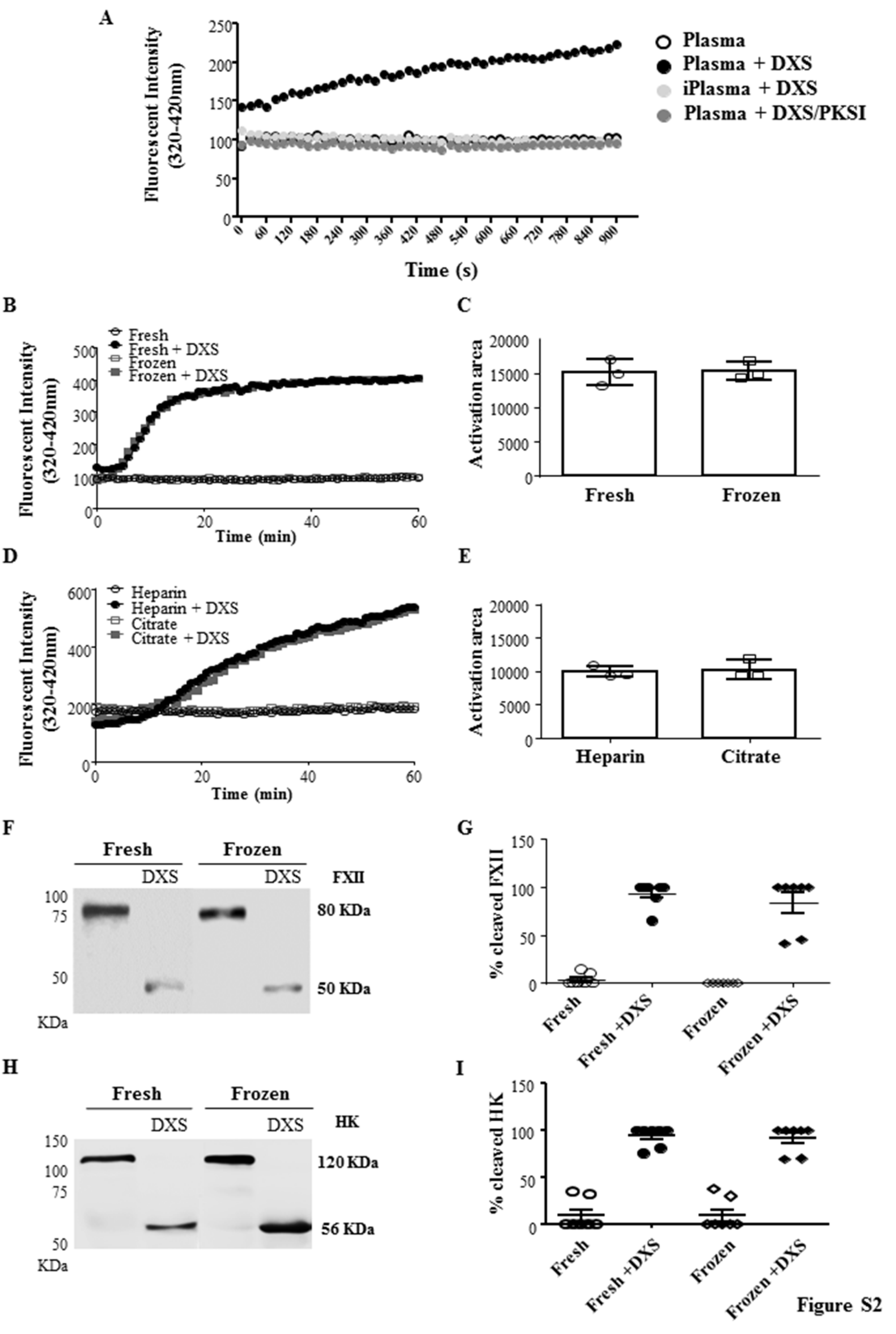

Figure S2. Evaluation of the interference of the plasmas stock condition for the hydrolysis assay.

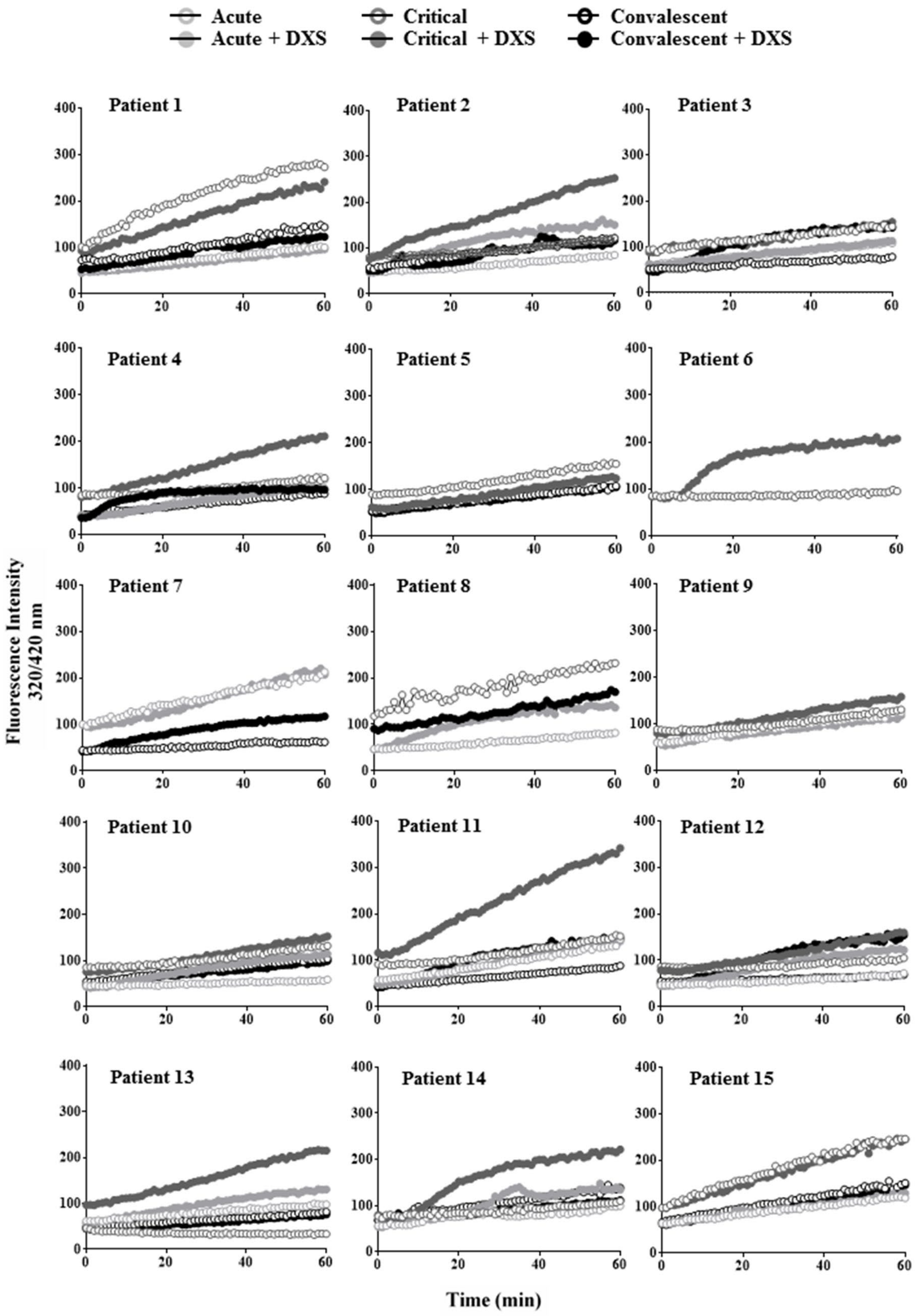
Acute
Acute + DXS
Critical
Critical + DXS
Convalescent
Convalescent + DXS
Patient 1
Patient 2
Patient 3
Patient 4
Patient 5
Patient 6
Patient 7
Patient 8
Patient 9
Patient 10
Patient 11
Patient 12
Patient 13
Patient 14
Patient 15
Fluorescence Intensity 320/420 nm
Time (min)

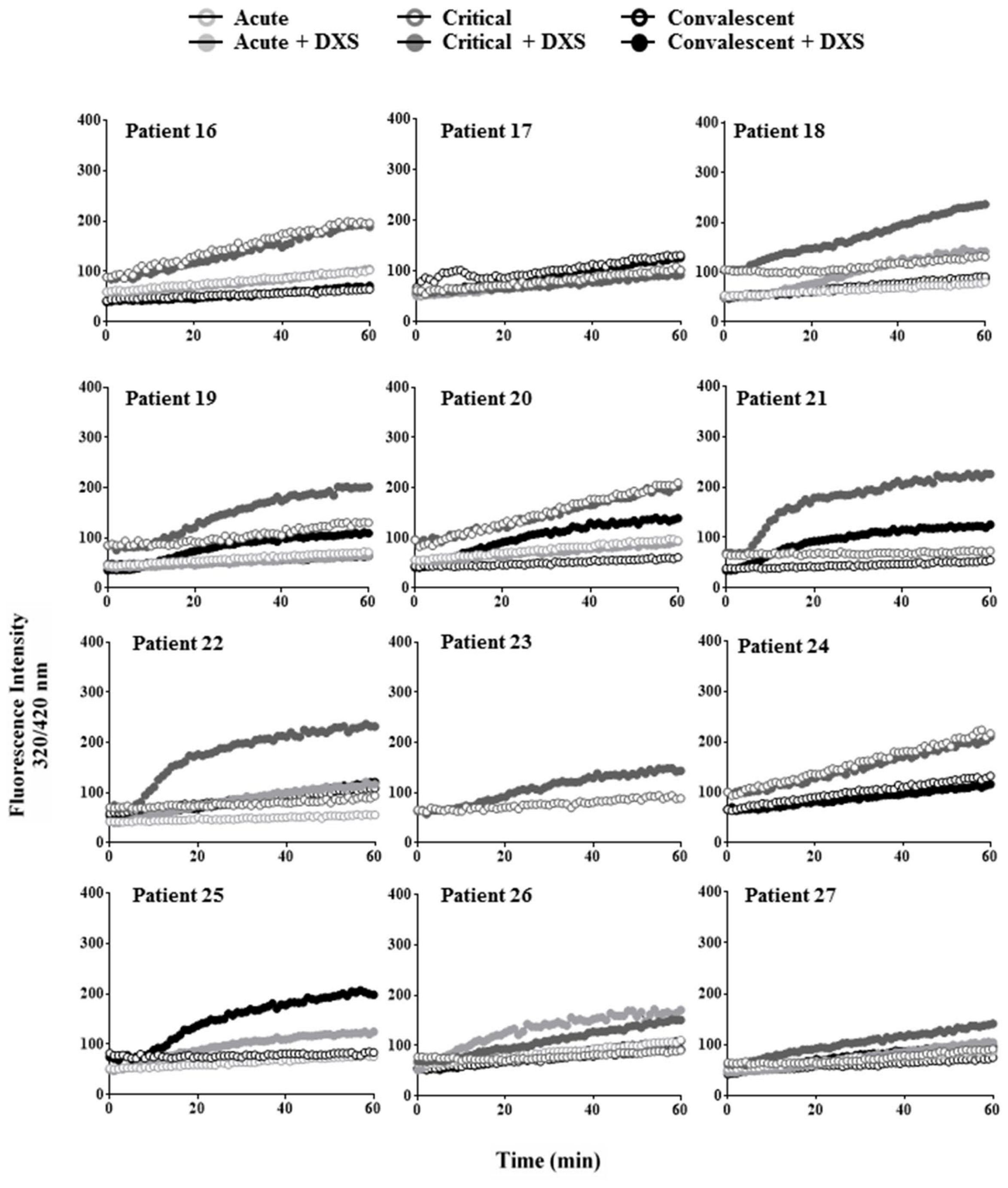

Figure S3. Hydrolysis of all DENV samples at different time points upon the symptom's onset.

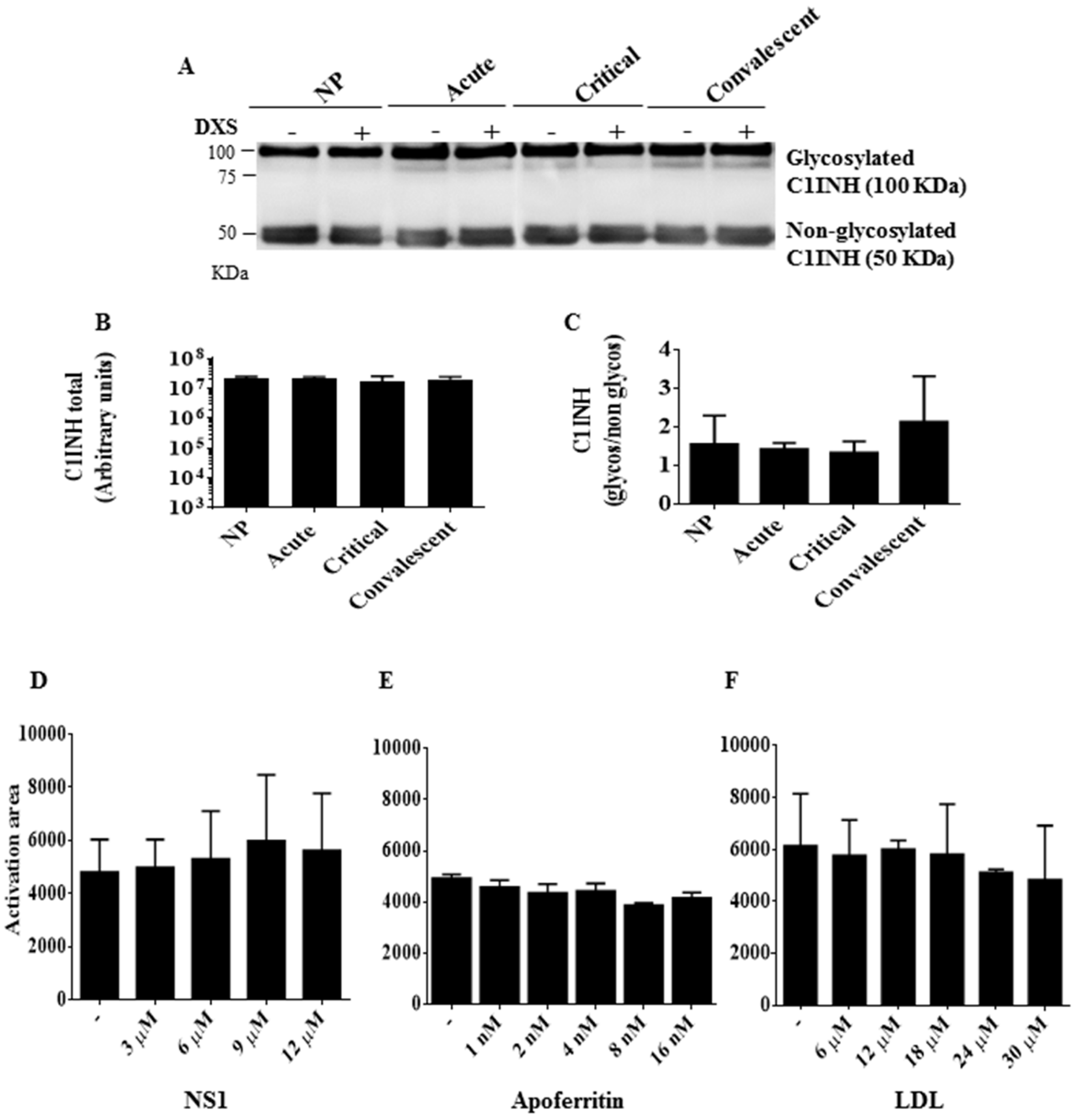

Figure S4. Lower DXS-driven KKS activation in the plasmas from dengue patients is not due to C1inhibitor, NS1, ferritinemia, or increased LDL.
